# Advances in microneedle Technology for Treatment of retinal degenerative diseases: a narrative review

**DOI:** 10.1007/s10544-025-00786-7

**Published:** 2026-01-15

**Authors:** Yuan Zong, Jiangman Liu, Mingming Yang, Jing Zhang, Yaru Zou, Zizhen Ye, Jiaxin Deng, Wendong Gu, Jingheng Du, Kyoko Ohno-Matsui, Koju Kamoi

**Affiliations:** 1 Department of Ophthalmology, Zhongshan Torch Development Zone People’s Hospital, Zhongshan, 528436 China; 2 Department of Ophthalmology & Visual Science, Graduate School of Medical and Dental Sciences, Institute of Science Tokyo, Tokyo, 113-8510 Japan; 3 International Ocular Surface Research Center, Institute of Ophthalmology, and Key Laboratory for Regenerative Medicine, Jinan University Medical School, Guangzhou, 510632 China; 4 Department of Ophthalmology & Visual Science, Graduate School of Medical and Dental Sciences, Institute of Science Tokyo, 1-5-45 Yushima, Bunkyo-ku, Tokyo, 113-8519 Japan

**Keywords:** Microneedles, Retinal degeneration, Drug delivery, Suprachoroidal injection, AMD, Diabetic retinopathy, Ocular therapeutics

## Abstract

**Abstract:**

Retinal degenerative diseases, such as age-related macular degeneration (AMD) and diabetic retinopathy (DR), present significant therapeutic challenges due to the complex anatomical and physiological barriers of the posterior eye. Conventional drug delivery methods, particularly intravitreal injections, are often limited by their invasiveness, rapid drug clearance, and burden on patient compliance. Microneedle technology has emerged as a paradigm-modifying approach for ocular drug delivery, offering a minimally invasive platform to bypass barriers like the blood-retinal barrier while targeting specific ocular tissues. This narrative review provides a critical overview of the latest advancements in microneedle technology for treating retinal degeneration, evaluating diverse configurations—including solid, hollow, dissolvable, coated, and hydrogel-forming designs—and their efficacy in facilitating suprachoroidal, intravitreal, and subretinal administration. Recent clinical trials highlighted in this review demonstrate promising results regarding safety, delivery efficiency, and patient acceptability. However, the translation from bench to bedside still faces hurdles in scale-up production, regulatory standardization, and long-term stability assessment. We discuss these technological challenges and explore future developments, such as the integration of smart materials and personalized approaches, emphasizing the potential of microneedle systems to revolutionize treatment paradigms through precise, controlled delivery to the posterior eye segments.

**Graphical abstract:**

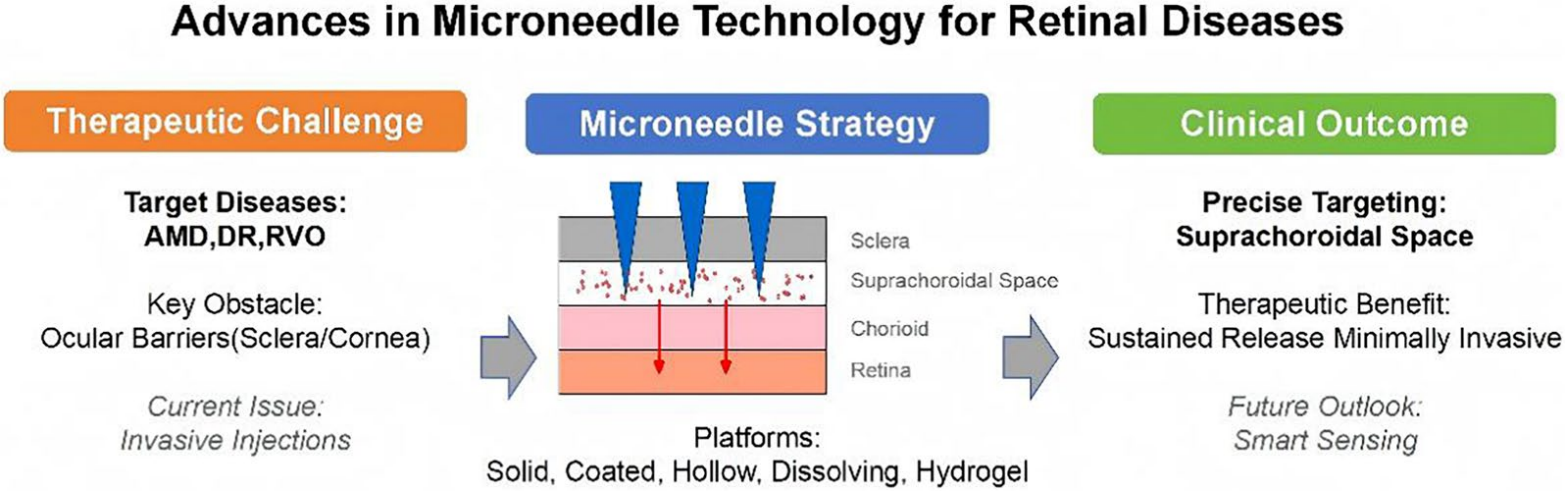

## Introduction

The structural integrity of retinal tissue is fundamental to visual function (So et al. 2025). Pathological retinal degenerative disorders, particularly age-related macular degeneration (AMD), diabetic retinopathy (DR), and diabetic macular edema (DME), pose significant threats to retinal homeostasis (Kovács-Valasek et al. 2023; Gowda et al. 2024a). Anti-VEGF therapeutics, which function by inhibiting the binding of VEGF to its receptors and consequently suppressing neovascularization, have emerged as the first-line treatment for these conditions (Zong et al. 2023). To minimize systemic side effects, localized and targeted delivery of anti-VEGF agents represents the preferred therapeutic approach for ophthalmic disorders. Microneedles have been recognized as an optimal delivery system for anti-VEGF drugs due to their ability to precisely administer therapeutic molecules into ocular tissues in a minimally invasive manner. This approach significantly reduces the risk of systemic adverse effects while enhancing patient compliance (Gowda et al. 2024b; Lee et al. 2018).

Current treatment approaches for posterior segment diseases primarily rely on intravitreal injections, which while effective, are associated with significant drawbacks including frequent administration requirements, risk of endophthalmitis, and poor patient compliance (Wu et al. 2024a; Lai et al. 2015). This has driven the search for alternative delivery strategies that can overcome these limitations while maintaining therapeutic efficacy.

Microneedles are minimally invasive drug delivery devices that consist of arrays of microscopic projections, typically shorter than 1 mm, designed to penetrate biological barriers in a controlled manner (Gowda et al. 2022a; Gowda et al. 2022b; Vora et al. 2023). These devices can be fabricated using various materials including metals (stainless steel, titanium), ceramics, silicon, and both biodegradable and non-biodegradable polymers, allowing for versatile drug delivery applications (Aldawood et al. 2021). In ocular drug delivery, microneedles offer several advantages over conventional methods, providing localized, efficient, and less invasive drug administration. Furthermore, polymeric dissolving microneedles can create micro-depots medication within ocular layers, enabling sustained drug release and potentially reducing the frequency of administration (Glover et al. 2023; Rojekar et al. 2024). This technology shows promise for overcoming the limitations of traditional ocular drug delivery methods, offering improved targeting and therapeutic efficacy for various ocular conditions.

This review aims to evaluate the latest advancements in microneedle technology for the treatment of retinal degenerative diseases, with a particular focus on engineering innovations, clinical applications, and therapeutic outcomes. To ensure a comprehensive analysis, we conducted a literature search using databases including PubMed, Web of Science, and Google Scholar. The search covered publications from 2000 to 2025, utilizing keywords such as ‘ocular microneedles’, ‘retinal drug delivery’, ‘suprachoroidal injection’, ‘microfabrication’, and ‘scleral biomechanics’. We prioritized studies that offer insights into engineering design, pharmacokinetic evaluations, and clinical translation, while excluding purely dermatological applications or non-English publications. Based on this framework, we examine various types of microneedles and their applications in different ocular delivery routes, specifically highlighting posterior segment drug delivery for retinal vascular disorders. Additionally, we analyze current challenges in clinical translation and discuss future perspectives of this promising technology.

## Posterior segment drug delivery routes

**Fig. 1:**
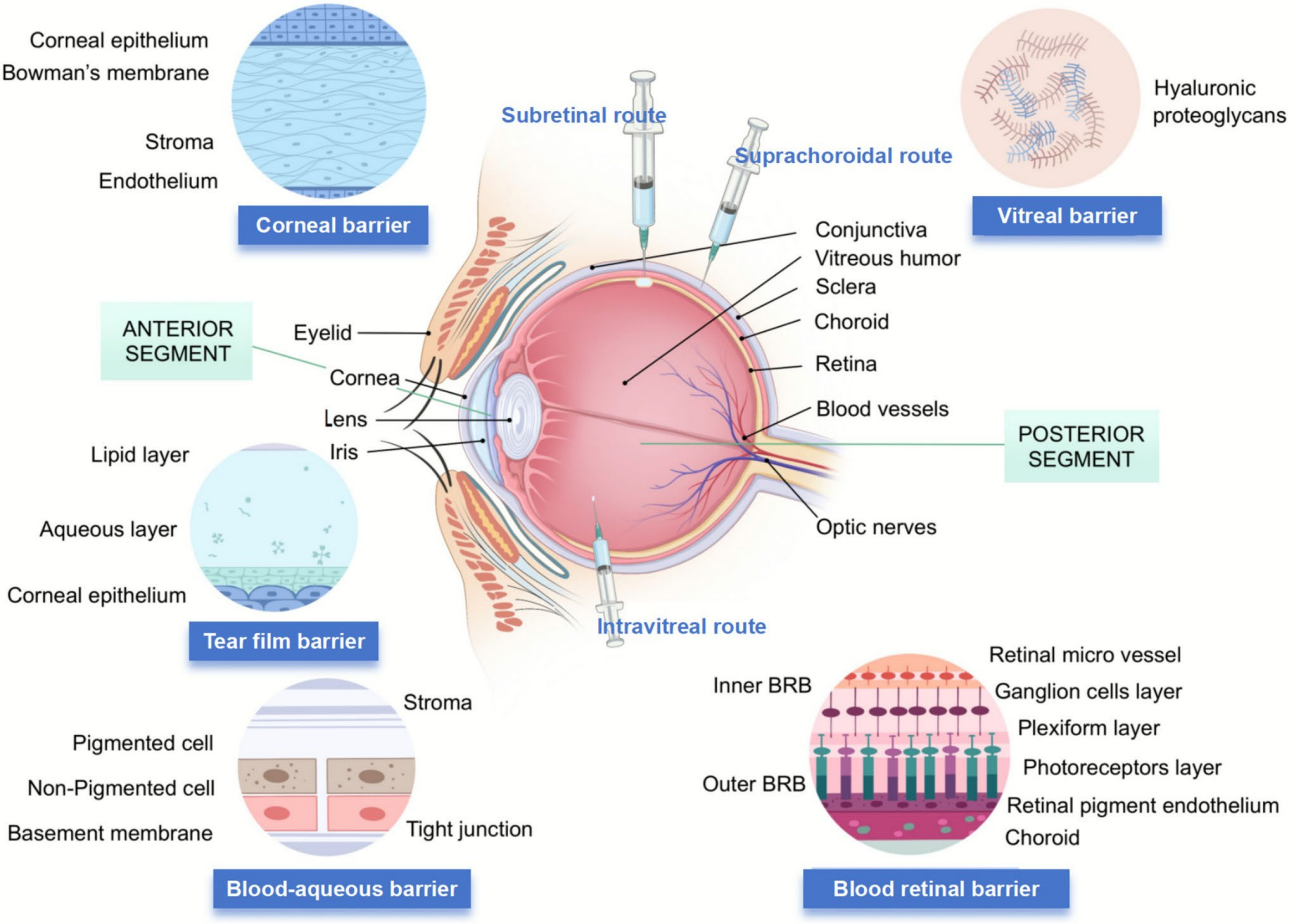
Schematic overview of ocular anatomy, physiological barriers, and drug delivery routes. The central diagram illustrates the anatomical structures of the anterior and posterior segments, highlighting intravitreal, suprachoroidal, and subretinal administration methods. Surrounding panels detail the microscopic composition of major ocular barriers, including the tear film, corneal, blood-aqueous, vitreal, and blood-retinal barriers. Created with BioGDP.com (Jiang et al. 2025)

The eye’s complex anatomical and physiological barriers pose significant challenges for drug delivery. Static barriers, including corneal epithelium, conjunctival epithelium, sclera, choroid, Bruch’s membrane, and retinal pigment epithelium, together with dynamic barriers such as choroidal and conjunctival blood flow, tear secretion, and lymphatic drainage, effectively reject foreign substances (Wang and Zhang 2023; Urtti 2006; Rodrigues et al. 2018). Figure 1 illustrates a schematic diagram of anatomical structure of the eye and the primary physiological barriers in ocular drug delivery.

For posterior segment diseases, intraocular drug delivery aims to achieve effective drug concentrations in target tissues while also considering patient compliance and minimizing invasiveness. Anatomical and physiological barriers are the main obstacles to delivering drugs to the posterior segment of the eye. Ocular drug delivery encompasses multiple administration routes, including topical, periocular, intravitreal, and subretinal/suprachoroidal approaches. The latter three modalities are particularly suitable for posterior segment therapeutics, as they bypass anterior anatomical barriers such as the tear film, corneal epithelium, and scleral tissue. These administration routes and their anatomical context are depicted in Fig. 1. This section will explore these posterior segment administration routes, analyzing their mechanisms, advantages, and limitations in clinical applications.

Intravitreal injection (IVT) represents the current standard of care for many posterior segment conditions, including neovascular age-related macular degeneration (nAMD), diabetic macular edema (DME), and retinal vein occlusion (Wu et al. 2023). The procedure involves a single-step insertion of a 30-gauge needle through the pars plana, approximately 3.5–4.5 mm posterior to the limbus, followed by direct injection into the vitreous cavity (Iqbal et al. 2023). By directly delivering drugs into the vitreous cavity, IVT circumvents both corneal/scleral barriers and the blood-retinal barrier (BRB), ensuring high bioavailability at the target site (Lai et al. 2015). However, this method has significant limitations. Many therapeutic drugs, especially hydrophobic compounds, have short half-lives, necessitating frequent injections (Wu et al. 2024a). Additionally, traditional IVT carries the risk of serious complications such as endophthalmitis, increased intraocular pressure, and cataract formation, thus requiring specialized surgical equipment and technically skilled operators (Wu et al. 2024a; Lai et al. 2015).

Subretinal injection (SR) offers unique advantages for specific applications, particularly gene therapy. The procedure requires a three-port pars plana vitrectomy followed by the creation of a localized retinal detachment through a controlled retinotomy. A specialized cannula is then used to slowly inject the therapeutic agent into the subretinal space (SRS), forming a characteristic bleb (Irigoyen et al. 2022). By delivering therapeutic agents directly to the outer retina through this approach, SR provides precise targeting of RPE and photoreceptor cells (Tripepi et al. 2023). However, SR is highly invasive, requiring specialized surgical procedures, and the distribution of the drug around the injection site is limited, which restricts its broader application (Wu et al. 2023).

Suprachoroidal injection (SC) has emerged as a promising alternative that balances efficacy with reduced invasiveness. The technique employs a specialized microneedle, typically 900–1100 μm in length, inserted perpendicular to the sclera 3–4.5 mm posterior to the limbus (Wu et al. 2023; Fazel et al. 2023). The procedure follows a distinct three-step process: initial needle insertion, gentle pressure application to form a seal, and slow injection over 5–10 seconds while maintaining position for an additional 3–5 seconds to prevent reflux. This approach enables drug accumulation in the choroid-sclera interface while bypassing various ocular barriers (Wu et al. 2023).

These limitations highlight unmet needs in the field of intraocular drug delivery, particularly in balancing invasiveness and treatment durability, as well as reducing surgical complexity. Against this backdrop, microneedle technology, with its minimally invasive penetration of barriers and controlled release capabilities, plays a particularly important role in addressing these challenges.

## Engineering microneedles for ocular application: Design criteria and biomechanical adaptations

**Fig. 2:**
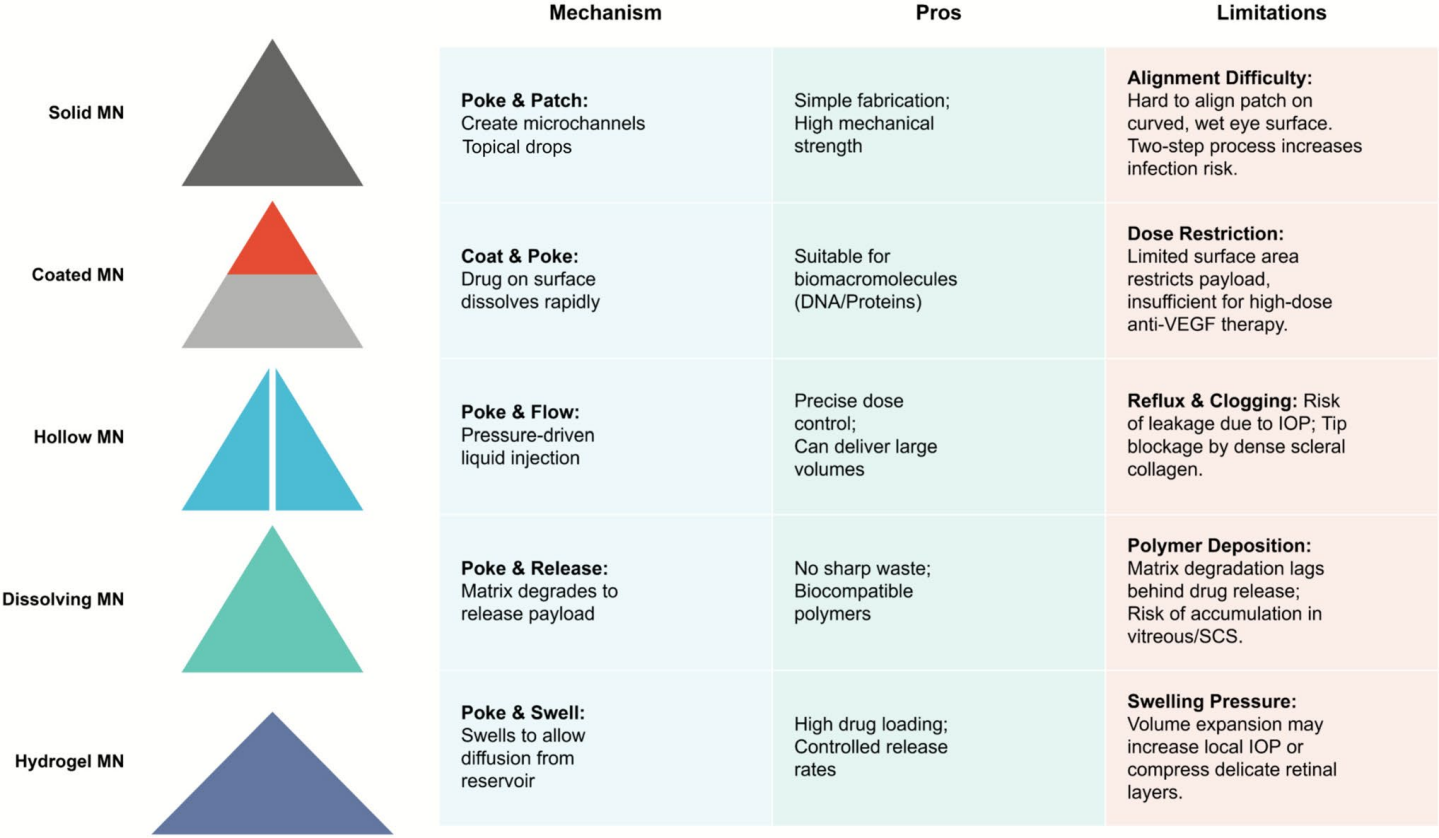
Comparative overview of microneedle (MN) platforms for ocular drug delivery. The schematic illustrates the structural classification of MNs (Solid, Coated, Hollow, Dissolving, and Hydrogel-forming) alongside a comprehensive analysis of their operational mechanisms, advantages, and application-specific constraints

Since their development in the early twenty-first century, Ophthalmic Microneedles have undergone more than 20 years of development and have become a promising ocular drug delivery platform. The lengths of currently developed ocular microneedles usually range from several hundred micrometers to less than 1 mm (Gadziński et al. 2022). They can be made of various materials and designed into different structures to meet specific therapeutic needs. According to their structural characteristics and drug delivery mechanisms, they can be roughly divided into five major categories: solid microneedles, hollow microneedles, dissolvable microneedles, coated microneedles, and hydrogel-forming microneedles. Figure 2 provides a comparative overview of these MN platforms for ocular drug delivery. The application of MNs in ophthalmology demands rigorous engineering adaptations to overcome the eye’s unique physiological barriers, which differ significantly from transdermal applications. The ocular surface presents specific challenges, including the “tenting effect” caused by the mobile conjunctiva, the dense collagen network of the sclera, and the rapid clearance of tear fluid (Rojekar et al. 2024; Shen et al. 2023). Consequently, MN design must prioritize mechanical strength for scleral penetration, dissolution kinetics matched to tear turnover, and biocompatibility to minimize inflammation (Rojekar et al. 2024; Bao et al. 2024). This section critically analyzes the fabrication methodologies, mechanical criteria, and specific configurations of ocular MNs. To provide a systematic evaluation of these diverse technologies, Table 1 summarizes the key engineering characteristics of the five primary ocular microneedle platforms. This comparative analysis highlights critical design parameters, including material selection (e.g., silicon vs. biodegradable polymers), geometric specifications tailored for scleral penetration (typically 500–1000 μmin length), and distinct drug release mechanisms. By juxtaposing the mechanical advantages of solid and hollow designs against the biocompatibility and compliance benefits of dissolving and hydrogel-forming systems, this comparison aims to guide the selection of appropriate platforms for specific retinal indications.

**Table 1:** Comparative Engineering Characteristics of Microneedle Platforms for Ocular Posterior Segment Delivery

MN Type	Common Materials	Typical Dimensions	Mechanism	Key Advantages	Major Limitations	Target Applications
Solid	Silicon, Stainless steel, Polymers (e.g., PLA)	Length: 500–1000 μm Tip radius: 1–20 μm	**Poke-and-patch:** Creates micropores for subsequent drug application.	High mechanical strength; Precise channel depth; Reusable (metals).	Two-step process (low compliance); Infection risk; No inherent drug payload; Requires removal.	Pre-treatment for large molecules (proteins, genes) and nanoparticles.
Coated	**Core:** Si, Steel, PLA **Coating:** PVA, PVP, HA, PLGA	Length: 500–1000 μm Coating thickness: 10–50 μm	**Coat-and-poke:** Coating dissolves quickly upon insertion.	Single-step operation; High core strength; Rapid delivery of potent drugs.	Limited drug payload (surface only); Risk of coating detachment; Uniformity control.	Potent therapeutics (anti-VEGF, DNA/AAV vectors), vaccines.
Hollow	Silicon (DRIE), Glass, Metals, 3D-printed resin	Length: 500–1000 μm (SCS specific) ID: 50–150 μm	**Flow injection:** Pressure-driven liquid infusion.	Precise dose/rate control; Large volume delivery; Handles high viscosity formulations.	Risk of clogging; Complex fabrication; Requires external fluidic device; Potential reflux.	Liquid formulations, gene therapy suspensions, SCS injection.
Dissolving	HA, PVA, PVP, PLGA, Chitosan, CMC-Na	Length: 500–1000 μm Tip radius: 1–20 μm	**Poke-and-release:** Polymer matrix degrades/dissolves to release payload.	No sharp waste (biodegradable); High patient compliance; Tunable release (rapid or sustained).	Lower mechanical strength (buckling risk); Hygroscopic stability issues; Polymer deposition in tissue.	Small molecules, sustained-release proteins, polymer-encapsulated drugs.
Hydrogel	Crosslinked HA, PVA, GelMA, PEGDA	Length: 500–1000 μm High swelling ratio	**Swell-and-diffuse:** Swells to form a conduit for diffusion.	High loading capacity (reservoir); Controlled/responsive release; Can extract ISF for diagnosis.	Slower onset of action; Mechanical strength in dry state; Swelling kinetics control.	Long-term therapy (glaucoma, AMD), Theranostics (e.g., glucose monitoring).

*MN* Microneedle; *ID* Inner Diameter; *SCS* Suprachoroidal Space; *HA* Hyaluronic Acid; *PLGA* Poly(lactic-co-glycolic acid); *ISF* Interstitial Fluid

### Microfabrication techniques and biomechanical integration

The fabrication of ocular MNs has transitioned from traditional subtractive manufacturing to high-precision additive methodologies, each offering distinct advantages for structural fidelity and material compatibility.

#### Micro-electro-mechanical systems (MEMS)

Traditional silicon-based MNs predominantly utilize Deep Reactive Ion Etching (DRIE), a core MEMS technology capable of producing high-aspect-ratio structures with vertical sidewalls and ultra-sharp tips (Xu et al. 2025; Bagolini et al. 2025; Huff 2021). This process ensures the resulting MNs possess a high Young’s modulus, necessary to withstand the axial loads encountered during scleral penetration. Recent innovations have further refined this through hybrid lithography approaches—integrating electron beam and ultraviolet nanoimprint lithography—to minimize feature deviation in complex composite structures (Xu et al. 2025). However, the inherent rigidity of silicon and the requisite cleanroom processing limit the cost-effectiveness and biodegradability of MEMS-fabricated devices.

#### Polymer micromolding and 3D printing

To address the material limitations of silicon, micromolding of biocompatible polymers (e.g., PLA, PVA, PLGA) has become ubiquitous. While cost-effective, conventional molding struggles with the replication of complex re-entrant structures or extremely high aspect ratios (Aldawood et al. 2021). High-precision 3D printing has emerged as a transformative alternative. Stereolithography (SLA) and Digital Light Processing (DLP) now achieve tip radii between 20 and 40 μm, offering a balance between resolution and production speed (Krieger et al. 2019). For sub-micron precision, Two-Photon Polymerization (TPP) allows for the direct fabrication of nanoscale features beyond the diffraction limit, enabling the creation of intricate, bio-inspired geometries that traditional lithography cannot achieve (Han et al. 2024).

#### Biomechanical design criteria

The mechanical performance of ocular MNs is governed by the interplay between insertion force (F_ins_) and fracture force (F_frac_). A safety margin where F_frac_ ≫ F_ins_ is prerequisite for reliable scleral micropuncture (Haider et al. 2024). The tough, elastic nature of the sclera necessitates optimization of the needle geometry to minimize insertion resistance. Bio-inspired designs have proven particularly effective in this domain. For instance, mimicking the jagged fascicle of the mosquito proboscis or incorporating barbs can significantly reduce penetration resistance and enhance tissue adhesion strength by up to 18-fold compared to smooth counterparts (Han et al. 2020). Similarly, beveled tip designs, fabricated via specialized MEMS protocols, facilitate smoother fluid extraction and reduce the axial force required for insertion (Bagolini et al. 2025).

### Solid microneedles

Solid MNs are primarily employed in “poke-and-patch” strategies, creating microchannels in the sclera or cornea to facilitate the subsequent permeation of topical formulations (Rojekar et al. 2024; Kulkarni et al. 2023). During the insertion process, solid microneedles are subjected to various forces, including bending force, lateral force, buckling force, axial force, and resistance. Thus, to penetrate the sclera without causing excessive deformation, the geometry and tip radius of the needle are critical determinants of performance. Research on penetration mechanics suggests that bio-inspired micro-nanostructures, such as mimicking mosquito mouthparts, can significantly reduce insertion force and improve stability during application (Kang et al. 2024). Inert materials like silicon or metals are often fabricated using Micro-Electro-Mechanical Systems (MEMS) technology to ensure a sufficient Young’s modulus, preventing buckling during insertion (Villarruel Mendoza et al. 2020; Mamun and Zhao 2022). However, due to the non-biodegradable nature of these materials, their application is typically transient to avoid long-term ocular irritation.

### Coated microneedles

Coated MNs consist of a solid core, typically metal or polymer, coated with a water-soluble drug formulation (Glover et al. 2023; Jiang et al. 2007). Upon insertion, the coating dissolves rapidly, leaving the solid core to be removed. This design is particularly suitable for delivering high-potency macromolecules, such as proteins and DNA, where dose requirements are low. The coating formulation must be engineered for instant solubility upon contact with interstitial fluid to ensure rapid release. However, the limited surface area of short ocular MNs restricts the total drug payload, necessitating layer-by-layer coating strategies or the use of highly concentrated formulations to achieve therapeutic levels (Glover et al. 2023).

### Hollow microneedles

Functioning as microscopic hypodermic needles, hollow MNs enable the direct infusion of liquid formulations, including solutions or suspensions, into specific compartments like the Suprachoroidal Space (SCS) or Subretinal Space (SRS) (Glover et al. 2023; Gade et al. 2024). For SCS delivery, needle length is precisely engineered, typically ranging from 900 to 1100 μm, to match the depth of the sclera-choroid interface. This route has been validated for delivering corticosteroids, such as triamcinolone acetonide, and gene therapy vectors like AAV, achieving high posterior segment concentrations while minimizing anterior side effects (Wu et al. 2023; Wu et al. 2024b). A major challenge with hollow MNs is the lack of tactile feedback during manual insertion, which poses risks of retinal perforation or reflux. Recent advancements addressing this issue have integrated force-sensing robotics with hollow MNs. For instance, a robot-assisted system demonstrated a 90% reduction in average puncture force and more stable trajectories compared to manual techniques, significantly improving the safety profile for precise subretinal injections (Wang et al. 2025). Furthermore, their diminutive dimensions render them susceptible to clogging and fracture when contrasted with conventional hypodermic needles (Glover et al. 2023).

### Dissolving microneedles

Dissolving microneedles are made by incorporating drug molecules into biodegradable and biocompatible polymers such as polyvinyl alcohol (PVA), polyvinylpyrrolidone (PVP), hyaluronic acid, polylactic acid (PLA), polyglycolic acid (PGA), and poly(lactic-co-glycolic acid) (PLGA) (Al-Nimry and Abu 2024; Dabholkar et al. 2021). In the context of ocular engineering, material selection is critical to balancing mechanical strength for insertion with precise dissolution kinetics. For instance, PLGA—a copolymer already validated in FDA-approved implants like Ozurdex®—offers tunable degradation rates achievable by adjusting the lactide-to-glycolide ratio and molecular weight, making it ideal for sustained posterior segment delivery (Shelley et al. 2022). To enhance patient compliance and ease of application, these systems are often designed for simple administration similar to contact lens insertion. A key engineering adaptation for the eye is the “detachable tip” mechanism: upon application, the drug-loaded tips remain embedded in the tissue to dissolve or degrade, while the backing substrate is removed, preventing residual waste and improving comfort (Glover et al. 2023; Al-Nimry and Abu 2024).

**Fig. 3:**
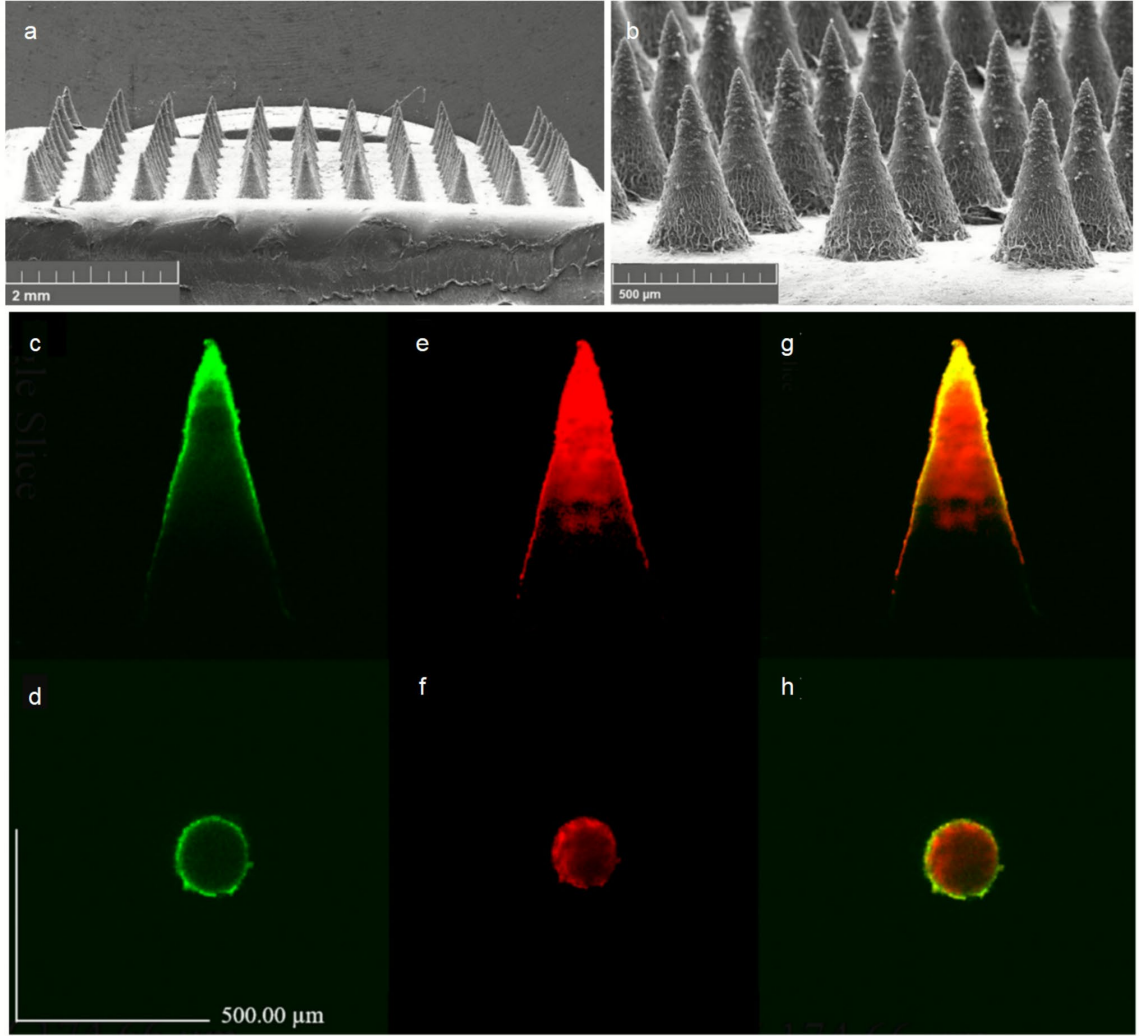
Morphological characteristics of fluconazole-microemulsion (FLUZ-ME)-loaded bilayer dissolving microneedles: (**a**, **b**) Scanning electron microscopy (SEM) images acquired at 30× and 130× magnification, respectively; (**c**, **d**) Confocal microscopy images (side and top views) demonstrating fluorescein sodium (FS) distribution (green fluorescence); (**e**, **f**) Rhodamine B localization (red fluorescence); (**g**, **h**) Composite visualization of FS (first layer) and rhodamine B (second layer) deposition patterns. Figure 3 was adapted from Suriyaamporn et al. (Suriyaamporn et al. 2022), distributed under the terms of the Creative Commons Attribution 4.0 International License (https://creativecommons.org/licenses/by/4.0/)

Advanced structural designs have been developed to achieve complex release profiles required for retinal therapies. Layered polymer architectures, such as those combining PLGA and PVP, enable controlled biphasic drug delivery (Park et al. 2019). This versatility extends to both anterior and posterior segment applications. For example, bilayer MNs incorporating microemulsion technology have significantly enhanced the penetration of fluconazole for fungal infections (Suriyaamporn et al. 2022) (Fig. 3). Furthermore, detachable biodegradable tips injected into the corneal stroma have shown efficacy in alleviating corneal opacity in Acanthamoeba keratitis models after a single injection, demonstrating the potential for localized, long-term release (Lee et al. 2018).

Despite these advancements, dissolving MNs face inherent engineering limitations that must be addressed. The physical constraints of ocular anatomy limit the needle height to typically less than 1 mm, which restricts drug loading capacity and necessitates the use of highly potent therapeutics (Glover et al. 2023). Furthermore, the stability of water-soluble polymers like PVA can be compromised by high humidity (Al-Nimry and Abu 2024), while biodegradable polymers like PLGA require prolonged retention at the administration site to ensure complete degradation (Rojekar et al. 2024). Finally, the geometric mismatch between the flat MN array and the curved ocular surface remains a challenge, potentially leading to uneven insertion depth, particularly at the array edges (Glover et al. 2023).

### Hydrogel-forming microneedles

Unlike dissolving MNs, hydrogel-forming MNs swell upon contact with ocular fluids, creating a porous conduit for continuous drug diffusion from a backing reservoir (Tucak et al. 2020; Fang et al. 2021). Crosslinked polymers, such as Alginate-Albumin scaffolds, can be engineered to control the release rate of therapeutics like fenofibrate, addressing the need for long-term management of RPE dysfunction (Abedin Zadeh et al. 2023). The integration of stimuli-responsive materials represents the next frontier in this field. Light-responsive, pH-sensitive, or ROS-responsive systems can trigger drug release in response to the pathological microenvironment of retinal diseases (Yang et al. 2024). For example, bio-inspired or sensor-integrated MNs could potentially monitor biomarkers, such as glucose or inflammatory cytokines, and adjust drug release accordingly, paving the way for closed-loop ocular therapeutics (Cicha et al. 2022).

**Fig. 4:**
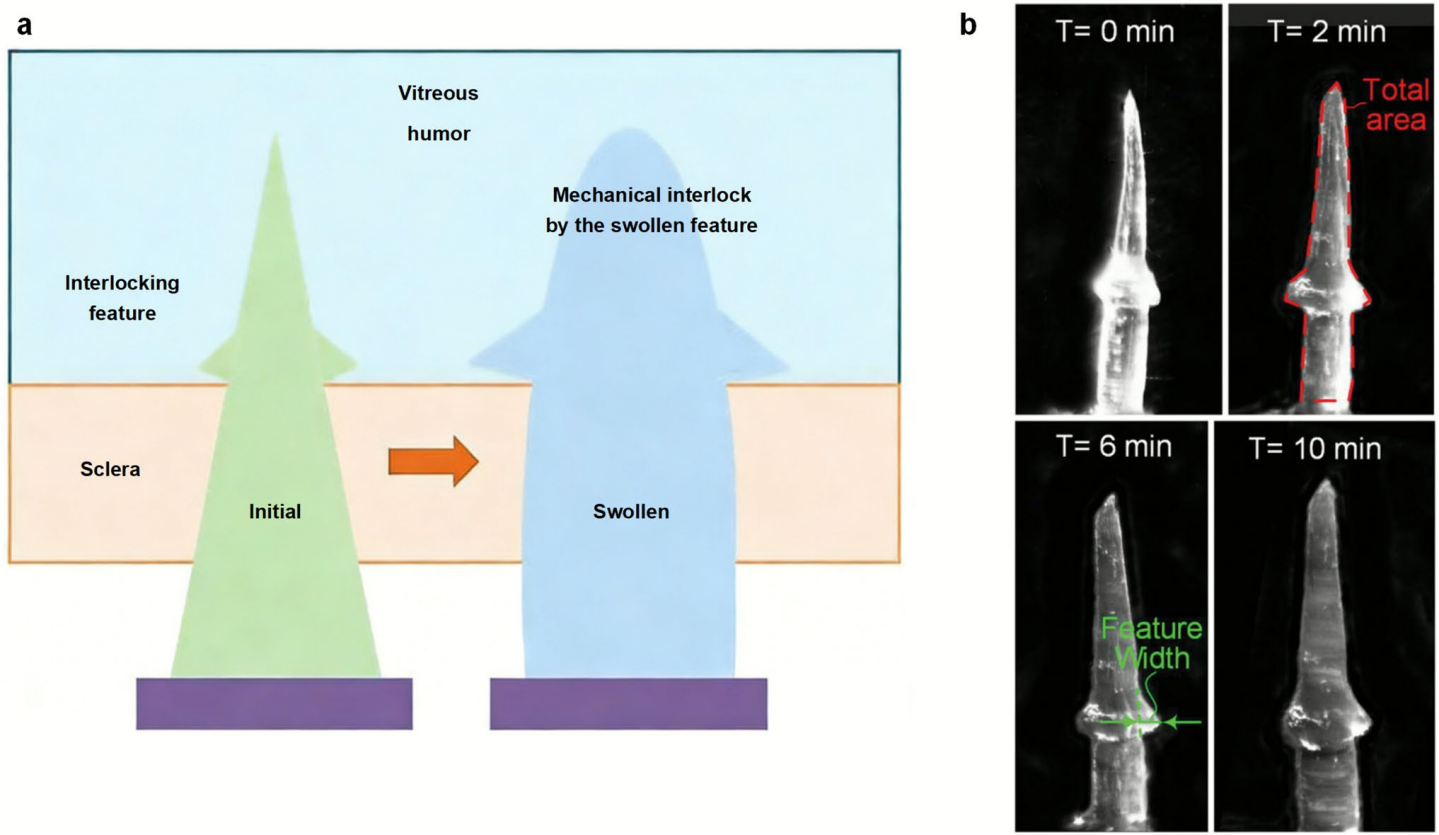
Mechanism and demonstration of the self-locking microneedle (MN). **a** Schematic illustration of the self-adhesive MN with interlocking features developed by Amer and Chen for sustained ocular drug delivery. The green structure represents the MN in its initial dry state penetrating the sclera. Upon contact with the vitreous humor, the MN absorbs fluid and transitions to a swollen state (blue), creating a mechanical interlock that secures the device in place. **b** Time-lapse optical microscopy images showing the morphological changes and progressive swelling of the MN immersed in a vitreous humor-mimicking gel at 0, 2, 6, and 10 minutes. The red dashed line (at T = 2 min) indicates the total area, and the green arrows (at T = 6 min) denote the feature width, which serve as key parameters for quantifying swelling kinetics. Adapted from Ref. (Amer and Chen 2020). Copyright 2020, John Wiley and Sons

Current research on the application of hydrogel-forming microneedles in ocular drug delivery remains limited. Maher Amer and Roland K. Chen developed a novel self-adhesive hydrogel microneedle system featuring an interlocking structure for sustained ocular drug administration (Amer and Chen 2020) (Fig. 4). Their findings demonstrate that this interlocking architecture undergoes swelling upon contact with biological fluids, resulting in an 80% enhancement in adhesion strength compared to conventional microneedles while maintaining comparable penetration capability. Furthermore, the research team has engineered a hydrogel-forming microneedle platform for the sustained and controlled ocular delivery of bevacizumab to treat AMD and DR, which will be discussed in detail in the following section.

## Therapeutic efficacy and pharmacokinetics in posterior segment delivery

### Overcoming ocular barriers and pharmacokinetic advantages

The eye’s complex anatomical barriers, including the corneal epithelium and the BRB, pose significant challenges for drug delivery. While intravitreal injections (IVT) bypass anterior barriers, they are invasive and subject to rapid clearance. MN technology offers a distinct advantage by creating micro-conduits that bypass the corneal epithelium and directly access the sclera or suprachoroidal space (SCS). Quantitative studies have recently validated this barrier-breaching capability. For instance, Sun et al. (Sun et al. 2024) reported that microneedle-assisted delivery of liposomes achieved an intraocular drug concentration 11.55 times higher than unmodified topical administration. Similarly, Fitaihi et al. (Fitaihi et al. 2023) quantified that transscleral MN delivery resulted in a 19.2% drug distribution in the vitreous humor, significantly exceeding that of passive diffusion. These data confirm that MNs can effectively overcome static ocular barriers to enhance posterior segment bioavailability.

### Microneedle-mediated Intravitreal injections (IVT)

IVT has long been the gold standard for treating most retinal degenerative diseases, such as nAMD and DME. Despite its widespread use, traditional IVT relies on relatively large-gauge needles, which pose inherent risks: drug reflux (where the injected agent leaks back through the needle tract) and direct damage to delicate ocular tissues like the retina or lens. MN technology addresses these mechanical limitations by offering a minimally invasive alternative that preserves ocular structural integrity.

A pioneering example is the tower microneedle (TM) developed by Lee et al. (Lee et al. 2013) specifically for intravitreal delivery. In their preclinical study, the research team injected phenylephrine—a sympathomimetic drug that induces pupil dilation—into rabbit eyes using TMs. Pupil size measurements confirmed that TMs achieved effective intravitreal drug delivery, as the drug reached therapeutic concentrations in the vitreous cavity. Histological analysis also showed that TMs caused far less tissue trauma compared to traditional subcutaneous injection needles: the smaller needle diameter and optimized tip geometry minimized disruption to the pars plana (the site of IVT insertion) and reduced the risk of retinal detachment or hemorrhage.

While TMs resolve the mechanical risks of traditional IVT, a more pressing limitation of standard IVT remains rapid drug clearance from the vitreous cavity. Veritti et al. (Veritti et al. 2025) emphasized that short drug retention directly undermines therapeutic efficacy, noting that even with anti-VEGF agents (the first-line treatment for retinal vascular diseases), clinicians must use high-dose formulations (e.g., 8 mg aflibercept) to extend treatment intervals. Without such adjustments, frequent injections (often every 4–8 weeks) are required to maintain drug levels—burdening patients and increasing long-term complication risks.

**Fig. 5:**
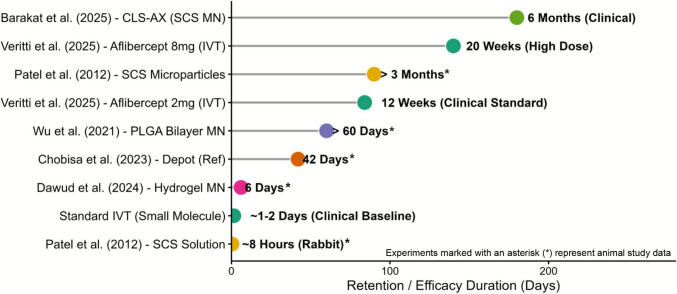
Comparison of ocular retention times between microneedle-based strategies and conventional intravitreal injections (IVT). Data represents a compilation of preclinical findings (Patel 2012 (Patel et al. 2012), Wu 2021 (Wu et al. 2021), Dawud 2024 (Dawud et al. 2024), Chobisa 2023 (Chobisa et al. 2023); derived from rodent/rabbit models) and recent clinical trial outcomes (Veritti 2025 (Veritti et al. 2025), Barakat 2025 (Barakat et al. 2025); human data), demonstrating the translational potential of microneedles to extend therapeutic duration from days to months. Animal Studies: yellow markers, and Clinical Trials: green markers. The X-axis has been unified to Days for all data points

MN technology offers a targeted solution to this clearance problem through a “drug depot effect.” By using dissolving or coated MNs, therapeutic agents can be embedded directly into the scleral tissue during insertion; as the MNs dissolve or degrade, the drug is gradually released into the vitreous cavity, rather than being rapidly cleared like liquid IVT formulations. Wu et al. (Wu et al. 2022) validated this mechanism in a key study: their rapid-dissolving bilayer MNs achieved an ovalbumin (a model protein drug) permeation rate of 86.99% ± 7.37% across the sclera—nearly double the rate of needle-free patches. This result proves that MN-based delivery creates a sustained drug reservoir in the ocular tissues, a capability standard liquid IVT cannot match. This depot effect–driven extension of ocular drug retention is most clearly demonstrated by quantitative comparisons, as shown in Fig. 5. For standard IVT, small-molecule drugs are cleared within just 1–2 days; even high-dose aflibercept (8 mg) only extends effective retention to 20 weeks (Veritti et al. 2025), which still requires 5–6 injections annually. In contrast, MN-based strategies drastically prolong drug action: SCS MNs delivering CLS-AX (axitinib suspension) maintained therapeutic efficacy for up to 6 months in clinical trials (Barakat et al. 2025), while (Wu et al. 2021). This extended retention directly translates to a reduced injection burden, for example, the 6-month efficacy of SCS MNs cuts annual injections from 6 to just 2.

### Microneedle-mediated Suprachoroidal injection (SCS)

The SCS serves as a natural potential space for targeted drug delivery to the choroid and retina. The pharmacokinetic behavior within the SCS is heavily dependent on the physicochemical properties of the therapeutic agent, a critical consideration for microneedle design.

Patel et al. (Patel et al. 2012) conducted a pivotal study using hollow microneedles to inject various formulations into the SCS. Their quantitative analysis revealed a size-dependent clearance mechanism: while small molecules cleared rapidly (half-life of 1.2 to 7.9 hours), microparticles ranging from 20 nm to 10 μm persisted in the SCS for months. This “depot effect” allows for sustained release directly adjacent to the choroidal neovascularization sites. Building on this, Gilger et al. (Gilger et al. 2013) demonstrated the clinical relevance of this route. In a porcine model of uveitis, microneedle-mediated SCS injection of triamcinolone acetonide (TA) proved superior to IVT. Notably, a low dose (0.2 mg) of TA delivered to the SCS was as effective as a high dose (2.0 mg) via IVT in reducing inflammation, while the low-dose IVT was ineffective. This 10-fold reduction in effective dose highlights the high bioavailability achieved by targeting the SCS. More recently, Hejri et al. (Hejri et al. 2023) optimized a high-precision hollow microneedle injector (160–260 μm length), achieving a 100% success rate in SCS delivery in rodent models with zero retinal perforations, further validating the safety profile of this route.

### Microneedle-mediated subretinal injection

Subretinal (SR) delivery allows for direct access to photoreceptors and retinal pigment epithelium (RPE) cells, making it the preferred route for gene therapy vectors like Adeno-associated virus (AAV). Conventional SR injection requires vitrectomy, a complex surgical procedure. Microneedles offer a “non-surgical” SR approach. Hejri et al. (Hejri et al. 2025) recently introduced a transscleral hollow microneedle strategy that enables SR delivery in less than one minute. In animal models, this method achieved safe, reliable delivery without vitrectomy, showing no significant retinal toxicity. This approach is particularly advantageous for delivering AAV vectors. Luo et al. (Luo et al. 2024) utilized a similar direct injection strategy to deliver a novel AAV capsid (AAVv128), demonstrating efficient transduction of RPE cells and significant suppression of Grade IV lesions in primate CNV models. By bypassing the vitreous, microneedle-mediated SR delivery minimizes the neutralization of viral vectors by pre-existing intravitreal antibodies, thereby enhancing transduction efficiency compared to IVT administration.

### Controlled release via dissolving and hydrogel microneedles

Beyond liquid injections, polymeric microneedles serve as self-implanting drug depots. The dissolution kinetics can be tuned by altering the polymer molecular weight. Thakur et al. (Thakur et al. 2016) showed that PVP-based microneedles dissolve within 10 to 180 seconds inside ocular tissue, rapidly releasing their payload. Conversely, Dawud et al. (Dawud et al. 2024) engineered hydrogel-forming microneedles that swell upon insertion, releasing 90% of their dexamethasone load over 6 days, demonstrating the versatility of MNs in managing treatment duration from minutes to weeks.

## Clinical trials of microneedle systems associated with retinal vascular degenerative diseases

Current clinical investigations of ocular microneedle delivery systems associated with retinal vascular degenerative diseases primarily focus on suprachoroidal administration. The most advanced research includes gene therapy and small molecule therapeutics, as summarized in Table 2.

**Table 2:** Overview of Ongoing Clinical Trials Using Microneedle Technology for Retinal Degenerative Disorders

Study Name	ClinicalTrials.gov ID	Phase	Microneedle Characteristics (Route)	Drug/Device	Indication	Sample Size	Key Safety Outcomes	Key Advantages of Microneedle Technology	Reference
ABBV-RGX-314 Clinical Program (ALTITUDE)	NCT04704921	Phase II	Microneedle-based SRS delivery	ABBV-RGX-314 (AAV vector)	DR without CI-DME	50 patients	Well tolerated; 3 cases of mild IOI resolved with steroids	70.8% achieved ≥1 step DRSS improvement; 89% reduction in vision-threatening events	(Glance n.d.; AbbVie 2025)
ABBV-RGX-314 Clinical Program (AAVIATE)	NCT04514653	Phase II	Microneedle-based SCS delivery	ABBV-RGX-314 (AAV vector)	Wet AMD	106 patients	Well tolerated; zero inflammation with prophylactic steroids (Cohort 6)	80% reduction in injection rate; 50% injection-free	(Glance n.d.; AbbVie 2025)
ABBV-RGX-314 Clinical Program (Fellow Eye)	NCT03999801	Phase II	Microneedle-based SRS delivery	ABBV-RGX-314 (AAV vector)	Wet AMD	9 patients	No inflammation without prophylactic steroids	97% reduction in treatment burden; 78% injection-free	(AbbVie 2025; Glance Editorial T 2024)
OASIS Trial	NCT04626128	Phase I/IIa	Microneedle-based SCS delivery	Single dose of axitinib injectable suspension	nAMD	27 patients	No drug dispersion to anterior chamber/vitreous	58% injection-free at 3 months; 57% extended to 6 months	(Barakat et al. 2025)
OASIS Extension	NCT05131646	Phase I/IIa Extension	Long-acting SCS microneedle	Single dose of axitinib injectable suspension	nAMD	14 patients	Stable safety profile through 6 months	90% reduction in treatment burden; 75% injection-free at 6 months	(Barakat et al. 2025)
PEACHTREE Study	NCT02595398	Phase III	Microneedle-based SCS delivery	Triamcinolone acetonide	Macular Edema secondary to Noninfectious Uveitis	160 patients	No serious AEs related to treatment; 11.5% with elevated IOP	47% gained ≥15 ETDRS letters; reduced CST mean reductions (153 μm versus 18 μm)	(Yeh et al. 2020)
Everads Injector Study	NCT06314217	Pilot	Microneedle-based SCS delivery (Everads Injector)	Triamcinolone acetonide	DME	10 patients	Mild transient hemorrhage; no severe AEs	Validated in-office use; circumferential macular distribution	(Modern Retina Editorial T. 2024; Everads 2025)

Abbreviations: *SCS* Suprachoroidal space, *SRS* Subretinal space, *AAV* Adeno-associated virus, *nAMD* Neovascular age-related macular degeneration, *IOP* Intraocular pressure, *ETDRS* Early treatment diabetic retinopathy study, *CST* Central subfield thickness, *DME* Diabetic macular edema

The ABBV-RGX-314 clinical program encompasses multiple Phase II trials evaluating this investigational gene therapy across different delivery methods and indications. The ALTITUDE trial (NCT04704921) investigated SRS delivery for diabetic retinopathy without CI-DME, enrolling 50 patients across two dose levels. One-year data showed that dose level 2 (5 × 10^11^ GC/eye) prevented disease progression in NPDR patients, with 70.8% achieving ≥1 step Diabetic Retinopathy Severity Scale (DRSS) improvement and an 89% reduction in vision-threatening events. The treatment was well-tolerated, with only three cases of mild intraocular inflammation that resolved with topical steroids (Glance n.d.). The AAVIATE trial, using SCS delivery in wet AMD patients, enrolled 106 patients across three dose levels. As of November 2023, the treatment demonstrated favorable safety with no drug-related serious adverse events. Notably, Cohort 6 (*n* = 21) at dose level 3 showed zero cases of inflammation when prophylactic topical steroids were used. The highest dose level achieved an 80% reduction in annualized injection rate, with 50% of patients remaining injection-free (Glance n.d.). The Fellow Eye sub-study evaluated SRS delivery (1.3 × 10^11^ GC/eye) in nine bilateral wet AMD patients. At 9 months post-administration, the study showed remarkable efficacy with a 97% reduction in treatment burden and 78% of patients remaining injection-free. No cases of intraocular inflammation were observed despite the absence of prophylactic steroids (Glance Editorial T 2024).

These combined results demonstrate ABBV-RGX-314’s potential as a promising gene therapy treatment across multiple retinal conditions, with both SCS and SRS delivery showing promising safety and efficacy profiles. Concurrently, the phase I/IIa OASIS trial investigated CLS-AX (axitinib suspension) in 27 treatment-experienced nAMD patients. This open-label study evaluated dose escalation via suprachoroidal administration, with doses ranging from 0.03 mg to 1.0 mg (Barakat et al. 2025). At 6-month follow-up, no serious adverse events or drug-related inflammation were reported. The trial demonstrated promising efficacy, with 58% of patients requiring no rescue therapy at 3 months and 57% remaining treatment-free at 6 months. Visual acuity and central subfield thickness remained stable throughout the study period (Barakat et al. 2025).

The PEACHTREE study is a Phase III study that evaluated the efficacy and safety of triamcinolone acetonide (CLS-TA) by microneedle-based SCS delivery for macular edema secondary to noninfectious uveitis (Yeh et al. 2020). This randomized trial enrolled 160 patients with either suprachoroidal CLS-TA or sham treatment. Results showed that 47% of patients in the CLS-TA arm gained 15 or more letters on the Early Treatment Diabetic Retinopathy Study (ETDRS) scale in best-corrected visual acuity (BCVA) at week 24, compared to 16% in the control arm (*p* < 0.001). Central subfield thickness (CST) was also significantly reduced in the CLS-TA group, with a mean reduction of 153 μm versus 18 μm in the control group (p < 0.001). The treatment was well-tolerated, with no serious adverse events related to the treatment reported. Elevated intraocular pressure (IOP) occurred in 11.5% of the CLS-TA group, which is lower than the typical rates observed with intravitreal corticosteroid treatments (Yeh et al. 2020). These findings highlight the potential benefits of microneedle-based SCS delivery in achieving targeted therapeutic effects with reduced systemic and anterior segment exposure. This has prompted the approval of this application by the FDA (Choi et al. 2025).

Performing intraocular injections without mechanical assistance imposes stringent technical demands on the operator. To address this challenge, specialized syringe-needle support devices have been developed recently to facilitate precise positioning and controlled delivery. Examples include the SCS microinjector (SCS™; Clearside Biomedical), guarded injection devices, and the InVitria® system (manufactured by FCI) (Hartman and Kompella 2017). The Everads injector, a microneedle device for SCS delivery, is currently under clinical investigation (NCT06314217) (Modern Retina Editorial T. 2024). This open-label pilot device study is evaluating the safety and performance of suprachoroidal triamcinolone acetonide injection in 10 patients with diabetic macular edema. The trial protocol includes comprehensive ophthalmic assessments over 6 weeks, encompassing visual acuity measurements, imaging studies, and safety monitoring. Preliminary results presented at the 2024 European Society of Retina Specialists (EURETINA) meeting confirmed the clinical feasibility of this technology, demonstrating rapid posterior pole drug distribution achievable in an outpatient setting (Modern Retina Editorial T. 2024). The study has completed enrollment of its initial patient cohort and is currently recruiting for subsequent cohorts.

Overall, these clinical trials demonstrate promising preliminary outcomes for microneedle-based ocular drug delivery systems. Of note is the favorable safety profile, with minimal reports of serious adverse events or inflammation across various trials and administration routes. Patient tolerance was excellent. Moreover, stable visual and anatomical outcomes were achieved while significantly reducing treatment burden for patients. These encouraging results suggest substantial clinical translation potential. However, prior to widespread clinical adoption, long-term follow-up data and larger patient cohorts are required to fully establish treatment durability and comprehensive safety profiles.

## Barriers and challenges in intraocular microneedle applications

The application of intraocular microneedles encounters multifaceted challenges, including time-consuming and costly microfabrication processes. The heterogeneous biomechanical properties of ocular tissues necessitate application-specific design optimization. Additionally, potential clinical complications associated with intravitreal drug delivery, such as intraocular hemorrhage, infection, and inflammation, present significant concerns (Glover et al. 2023).

### Sterile inflammation and immunological responses

Beyond the immediate risk of infectious endophthalmitis, the mechanical breach of ocular barriers inevitably perturbs the local immune microenvironment, precipitating sterile inflammation. The insertion trauma can induce force-induced efferocytosis impairment in resident immune cells, necessitating the development of immunomodulatory strategies—such as nitric-oxide driven nanomotors—to revitalize macrophage energy metabolism and mitigate inflammatory sequelae (Tan et al. 2025). Although certain biodegradable materials like silica demonstrate acceptable ocular tolerance in preclinical models (Winter et al. 2025), the biodegradation process itself may generate ROS or acidic byproducts that exacerbate local tissue stress. Consequently, the incorporation of antioxidant functionalities, such as nanozymes, into the microneedle matrix has been proposed to modulate the oxidative milieu and suppress pro-inflammatory pathways during the wound healing process (Hu et al. 2024).

### Manufacturing scalability and regulatory ambiguity

The translation from bench-top fabrication to commercial utilization is substantially impeded by Good Manufacturing Practice (GMP) compliance hurdles. While 3D printing and lithography offer precision at the laboratory scale, these techniques face significant challenges in achieving the high-throughput consistency and cost-efficiency required for mass production (Fu et al. 2024). A critical, often overlooked bottleneck is sterilization; conventional modalities such as gamma irradiation or ethylene oxide frequently compromise the structural integrity of polymeric microneedles or denature sensitive biologic payloads, underscoring the urgent need for novel, gentle sterilization protocols validated specifically for ocular combination products (Wu et al. 2024b).

Furthermore, regulatory frameworks struggle to keep pace with technological convergence. The classification of microneedles oscillates between “drug delivery systems” and “medical devices,” or “combination products,” creating a complex approval landscape that varies by jurisdiction (Bao et al. 2024). This regulatory uncertainty, coupled with the lack of standardized characterization metrics for microneedle geometry and penetration mechanics, creates significant commercialization barriers. The absence of universal quality evaluation standards further compounds the production scalability issue. Therefore, research institutions must engage early and consistently with regulatory bodies to clarify the approval pathway for clinical translation (Wu et al. 2024b; Lutton et al. 2015).

## Conclusions and future perspectives

Over the past two decades, microneedle technology has emerged as a potentially viable platform for ocular drug delivery, offering significant advantages in treating retinal degenerative disorders. This review has comprehensively examined various microneedle designs, including solid, hollow, dissolving, coated, and hydrogel-forming types, each with distinct characteristics suited for specific therapeutic applications. These systems have demonstrated remarkable progress in addressing the challenges of conventional ocular drug delivery methods, particularly in achieving precise targeting, controlled release, and enhanced patient compliance.

Current clinical trials, especially those utilizing SC delivery systems, have demonstrated considerable safety and efficacy in delivering therapeutics for conditions such as AMD and DR. These advances have significantly enhanced the possibility of outpatient treatment for retinal degenerative diseases, notably mitigating the burden on both patients and healthcare providers.

The field of ocular microneedle technology is poised for a paradigm shift from passive delivery vehicles to multifunctional ‘theranostic’ systems. By accessing the interstitial fluid (ISF), next-generation microneedles could facilitate continuous, minimally invasive monitoring of physiological biomarkers—such as glucose, lactate, or specific inflammatory cytokines—thereby offering a real-time window into ocular pathology (Gowda et al. 2024a; Lin et al. 2025). The convergence of microneedle technology with artificial intelligence and bioelectronics further augments this capability, enabling the analysis of complex biological signals to trigger precise, on-demand drug release (Ashraf et al. 2025; Sun et al. 2023).

However, several critical challenges must be addressed to fully realize the potential of this technology. These include optimizing manufacturing processes for large-scale production, establishing standardized regulatory frameworks, and developing more robust quality control measures. Future research should focus on improving the mechanical properties and drug loading capacity of microneedles while maintaining their safety profile. Additionally, long-term stability studies and comprehensive pharmacokinetic evaluations are needed to support clinical translation.

The convergence of advanced materials science, precision engineering, and biological understanding will be crucial in developing next-generation microneedle systems. As this technology continues to evolve, it holds the promise of providing more effective, less invasive, and more patient-friendly treatments for various ocular conditions. With continued research and development efforts, microneedle-based drug delivery systems are expected to play an increasingly important role in ophthalmology, potentially transforming the standard of care for retinal degenerative diseases and improving outcomes for millions of patients worldwide.

## Data Availability

Data sharing not applicable to this article as no datasets were generated or analysed during the current study.
